# Supplementary material for: Comparative Analysis of Plastomes in Elsholtzieae: Phylogenetic Relationships and Potential Molecular Markers
Source: Int J Mol Sci. 2023 Oct 17;24(20):15263. doi: 10.3390/ijms242015263 (PMC10607353; doi:10.3390/ijms242015263)
Supplement: Supplementary file 1 [file ijms-24-15263-s001.zip › Supplementary_files/Figure_Table_Tite.docx]

**Supplementary Figure S1.** The relationships among the sizes of complete plastome, LSC, SSC, and IR regions. (A–F) Correlations among each region.

**Supplementary Figure S2.** Colinear analysis of the 18 Elsholtzieae plastomes using MAUVE software. The annotated rRNA, protein-coding and tRNA genes are shown in red, white and green boxes, respectively.

**Supplementary Figure S3.** Comparison of junctions between the LSC, SSC, and IRs in the 11 plastomes of *Elsholtzia*. Distance in this figure is not to scale. Pseudogenes are marked by Ψ.

**Supplementary Figure S4.** Amino acid frequencies in nine Elsholtzieae plastomes based on protein-coding sequences.

**Supplementary Figure S5.** Distribution of simple sequence repeats (SSR) in the nine Elsholtzieae plastomes. (A) Number of SSRs in the LSC, SSC and IR regions. (B) Number of SSRs in the intergenic spacer (IGS), intron sequences, and protein-coding regions (CDS).

**Supplementary Figure S6.** The type and presence of repeated sequences in the nine Elsholtzieae plastomes. (A) Percentage of four repeat types; (B) Types and number of long repeats. (C) Number of repeats divided by length.

**Supplementary Table S1.** Complete plastome features of the 18 Elsholtzieae and two outgroups plastomes.

**Supplementary Table S2.** Codon usage within the protein-coding sequences of the 18 Elsholtzieae plastomes.

**Supplementary Table S3.** Statistics for length, nucleotide variability of coding region and intergenic regions of the 18 Elsholtzieae plastomes.
