# Supplementary figures and images for: Comparative Analysis of Plastomes in Elsholtzieae: Phylogenetic Relationships and Potential Molecular Markers
Source: Int J Mol Sci. 2023 Oct 17;24(20):15263. doi: 10.3390/ijms242015263 (PMC10607353; doi:10.3390/ijms242015263)

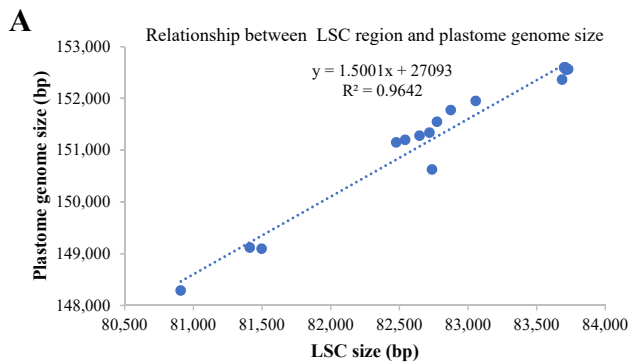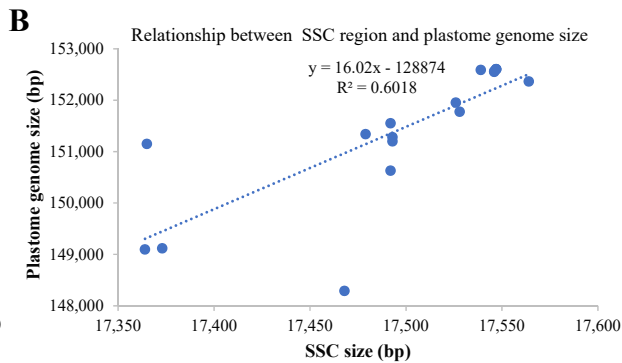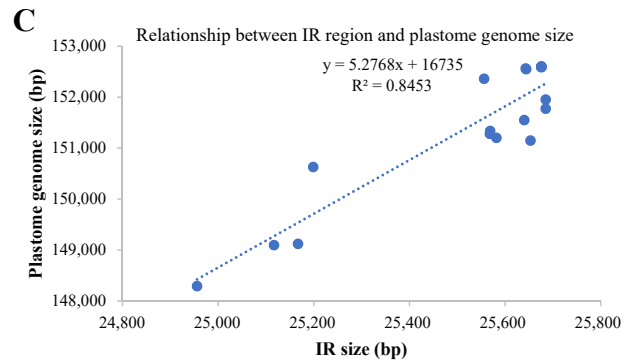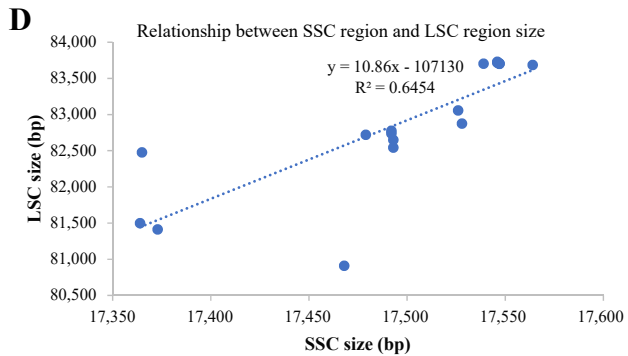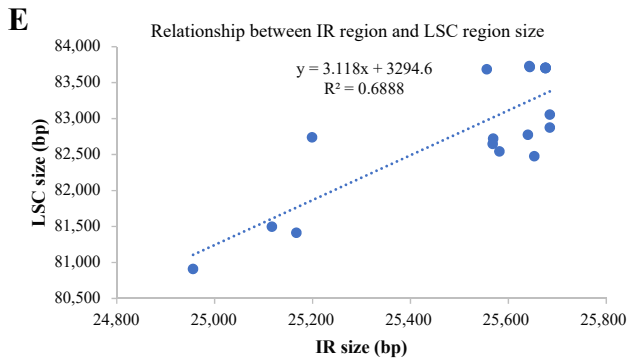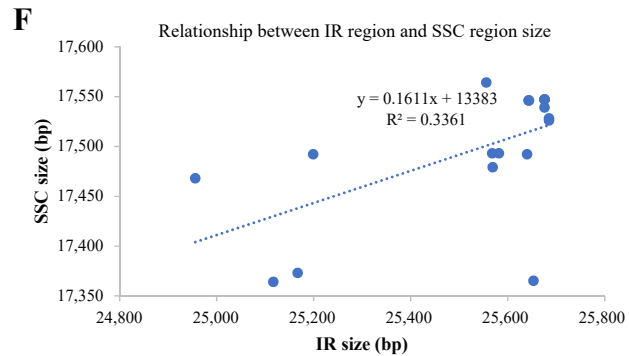

Supplement: Supplementary file 1 [file ijms-24-15263-s001.zip › Supplementary_files/Supplementary Figure S1.pdf]

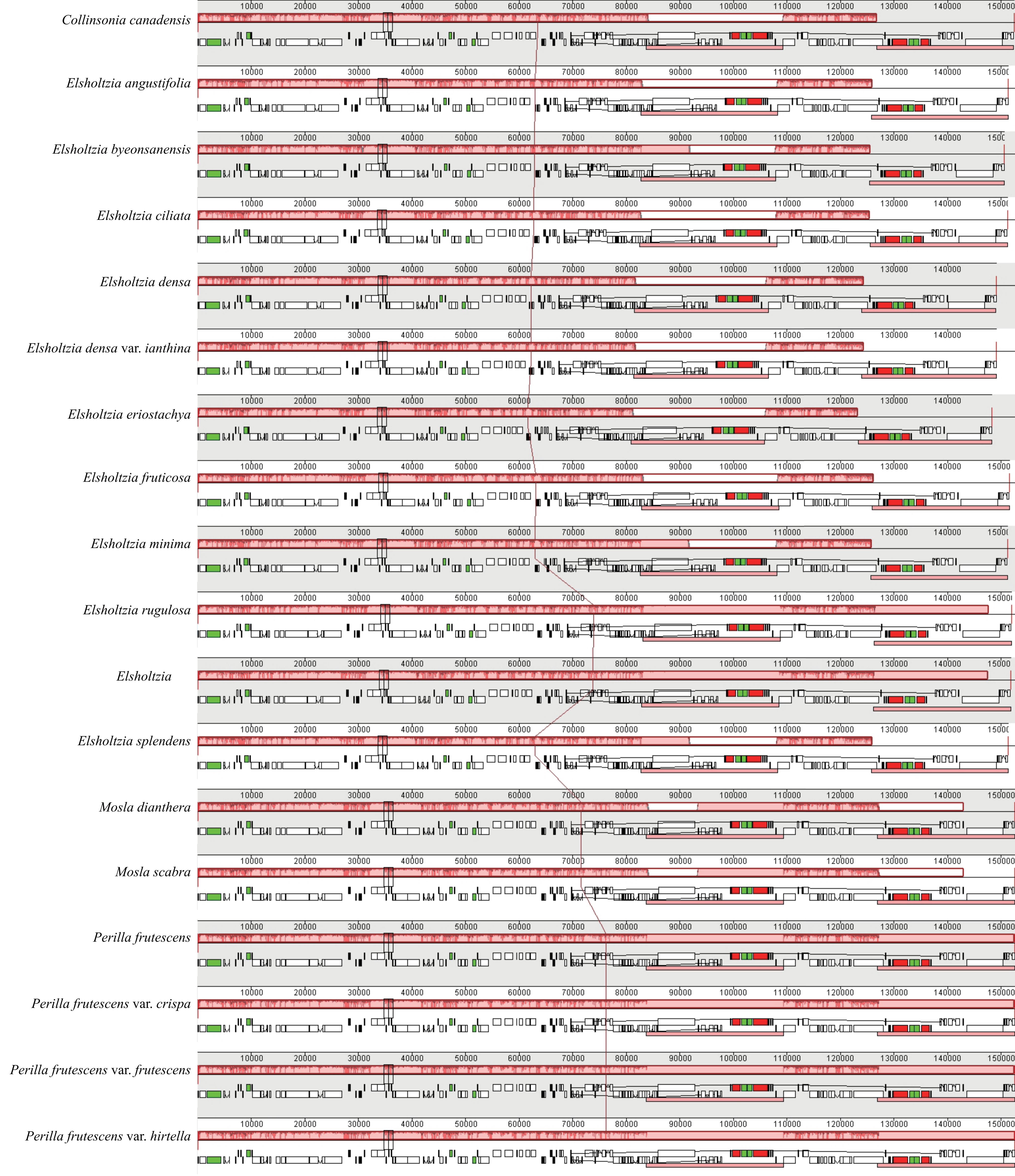

Supplement: Supplementary file 1 [file ijms-24-15263-s001.zip › Supplementary_files/Supplementary Figure S2.pdf]

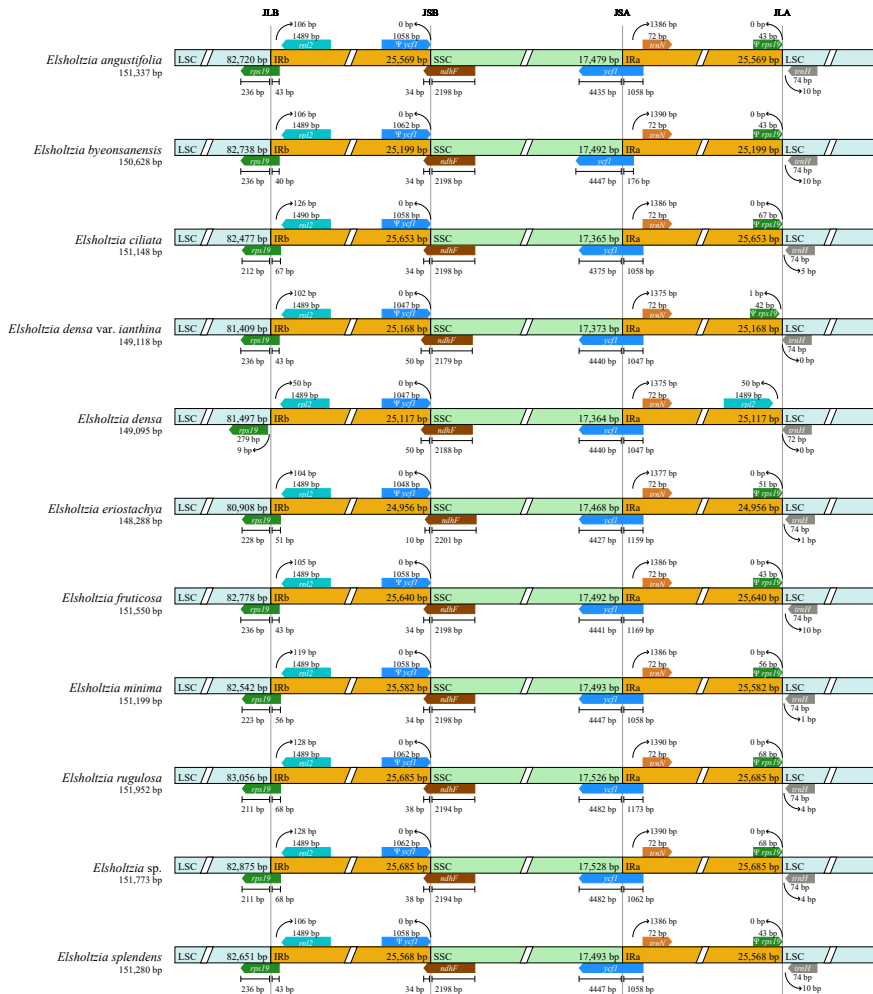

Supplement: Supplementary file 1 [file ijms-24-15263-s001.zip › Supplementary_files/Supplementary Figure S3.pdf]

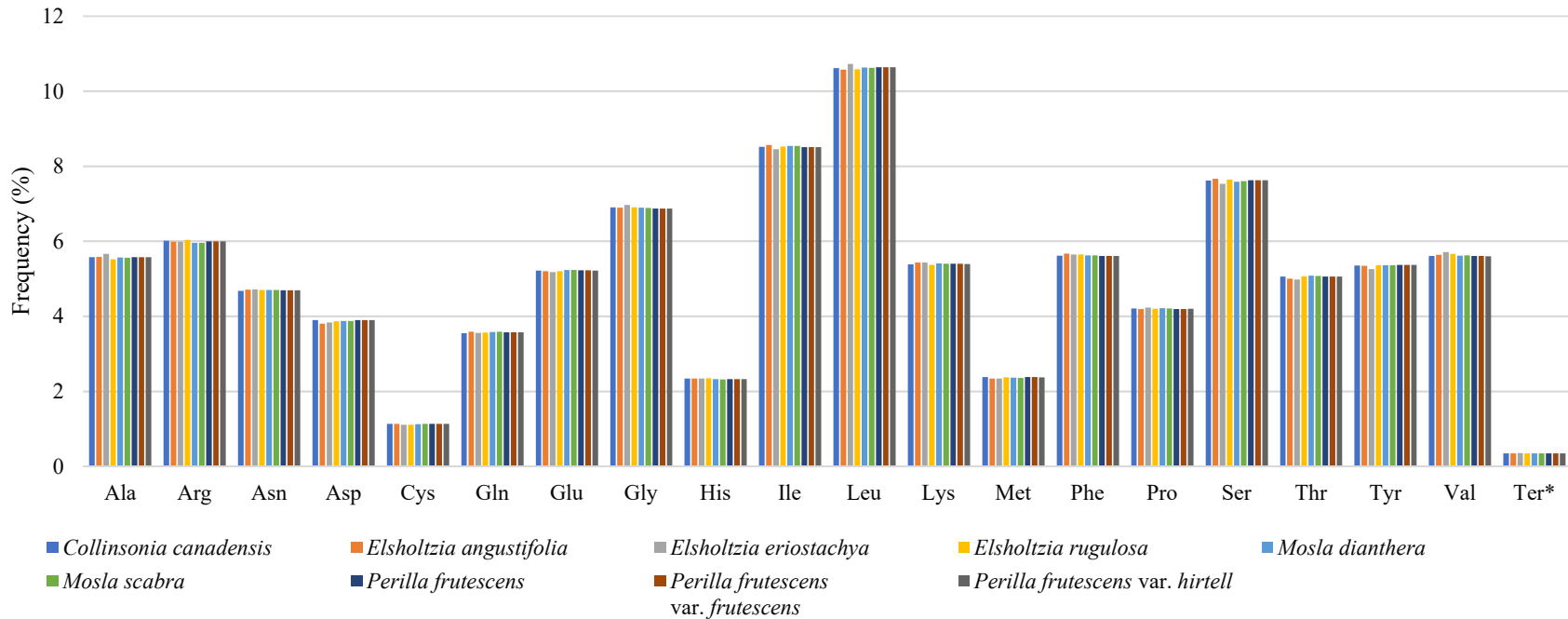

Supplement: Supplementary file 1 [file ijms-24-15263-s001.zip › Supplementary_files/Supplementary Figure S4.pdf]

A

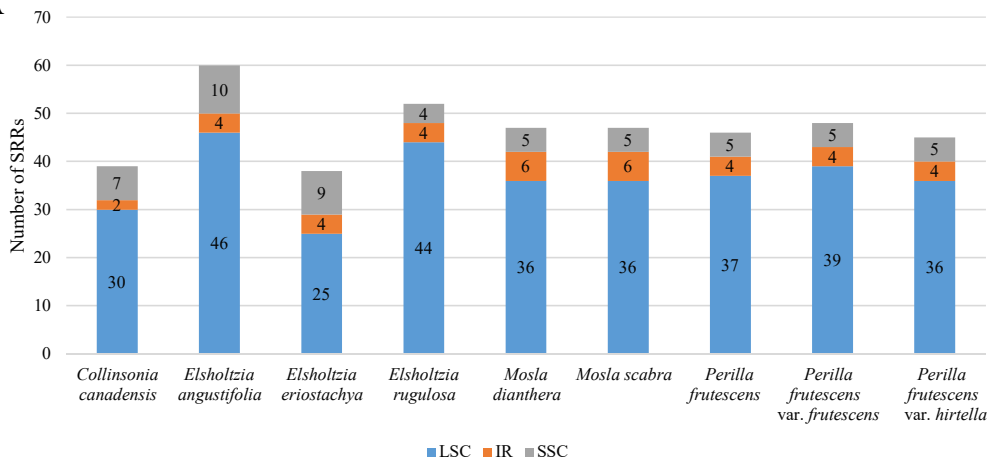

B

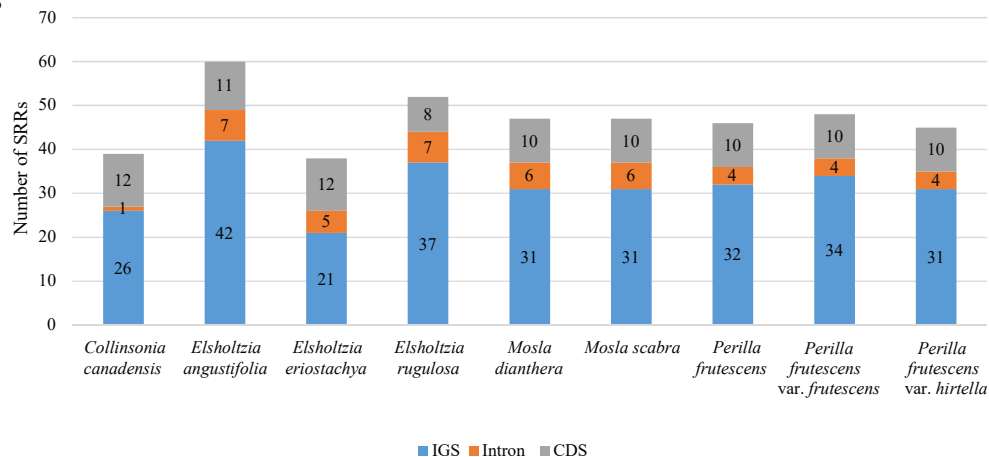

Supplement: Supplementary file 1 [file ijms-24-15263-s001.zip › Supplementary_files/Supplementary Figure S5.pdf]

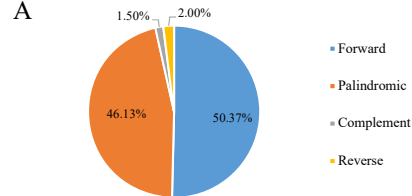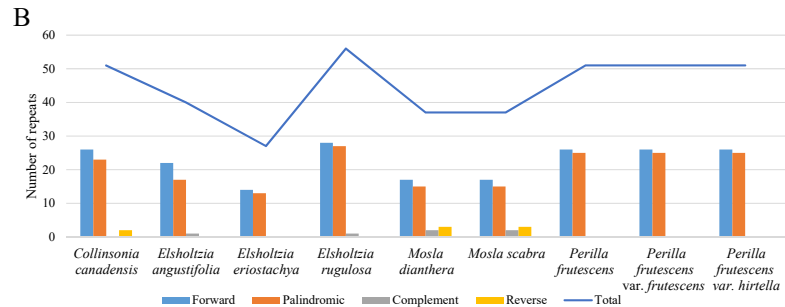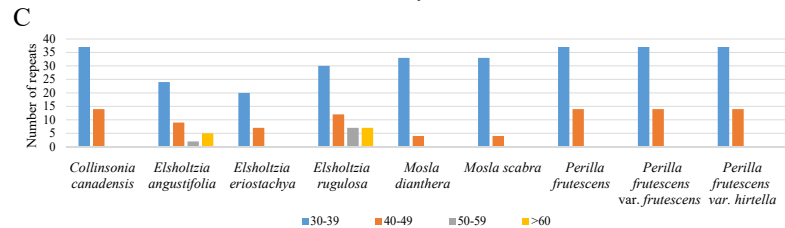

Supplement: Supplementary file 1 [file ijms-24-15263-s001.zip › Supplementary_files/Supplementary Figure S6.pdf]
